# Haemoptysis secondary to a complicated hydatid cyst of the lung: A case report

**DOI:** 10.7196/AJTCCM.2020.v26i2.029

**Published:** 2020-06-15

**Authors:** R Manganyi, A Moodley, T Pennel, C Ofoegbu, A Linegar

**Affiliations:** Chris Barnard Division of Cardiothoracic Surgery, Groote Schuur Hospital, University of Cape Town, South Africa

**Keywords:** Noonan syndrome, haemoptysis, polyps, bronchial artery

## Abstract

Pulmonary hydatid disease is almost exclusively caused by the infestation of the larval stage of Echinococcus granulosus. Humans are infected, accidentally, through the faeco-oral route by the ingestion of food and milk, contaminated by dog faeces containing the ova of parasites or direct contact with dogs. We describe an unusual cause of massive haemoptysis in a young male who had bilateral lung hydatid cysts as well as a large splenic hydatic cyst. He underwent bilateral thoracotomies for cyst excision for relief of haemoptysis.

## Background

The larvae of Echinococcus granulosus, also referred to as the tapeworm, is a common helminthic infection affecting carnivores such as dogs and wolves. It is known to cause disease in humans, who act as intermediate hosts after accidental infection by the eggs of the worm.^[1,2]^

## Case

**Fig. 1:**
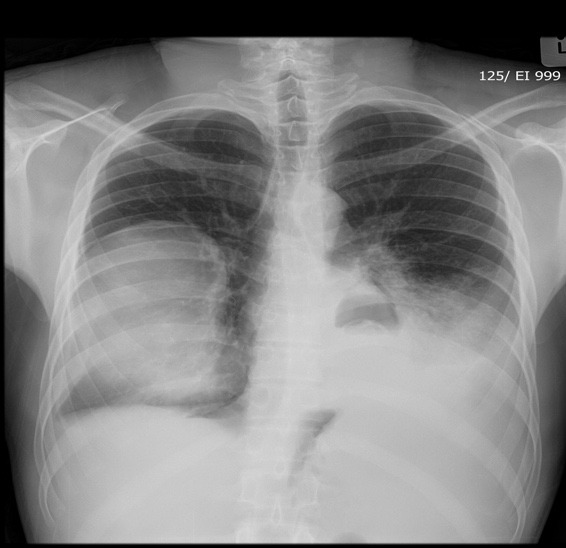
Chest radiograph at presentation

A 22-year-old male patient presented to the Emergency Department with a 2-day history of haemoptysis (~100 mL) and dysphagia. He had vital signs within the normal range, was haemodynamically stable and had no stigmata of any chronic medical conditions. A respiratory examination revealed decreased air entry in the right middle and lower zones. The chest radiograph showed a well circumscribed dense homogeneous opacity in the mid to lower zones of the right hemithorax and a small left lower zone cavity with a water-lily sign and obliteration of the left hemi-diaphragm (Fig. 1). He was suspected to have bilateral pulmonary hydatid cysts, with a complicated left cyst.

**Fig. 2:**
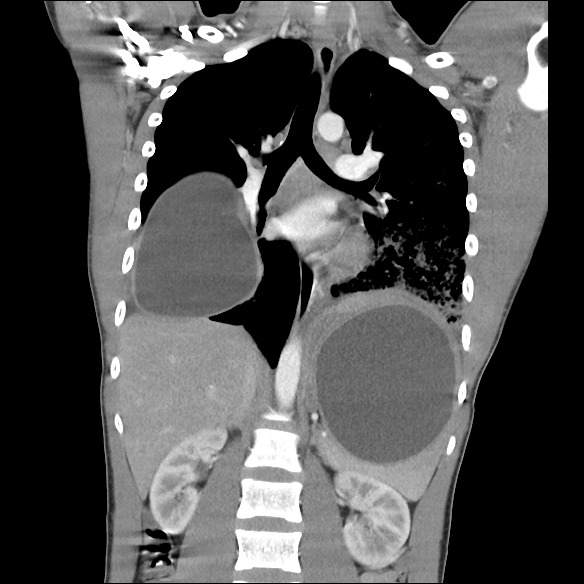
Computed tomography chest scan (axial view).

Contrast computed tomography (CT) chest scan revealed multiple cysts in both lungs (left side-complicated) and spleen consistent with hydatid disease. The right lower zone (RLZ) cyst measured 94 × 110 mm (axial), 149 mm (long) and the left lower lobe cyst was collapsed and measured about 48 mm. The spleen had a 127 mm cystic lesion with a normal looking liver (Fig. 2).

The patient was commenced on intravenous co-amoxiclav and oral albendazole and had subsequent cessation of haemoptysis and then referral for surgical management of the pulmonary and abdominal cystic lesions. He subsequently had an elective right thoracotomy and cystectomy with air leak closure without capitonnage at 6 weeks post presentation.

The operation revealed a large hydatid cyst that ruptured upon efforts to enucleate it. The membranes were delivered, major air leaks were closed with 3/0 prolene sutures and the cavity was left without capitonnage being performed. Routine chest closure was done with 2 drains placed and secured. The postoperative intensive care unit (ICU) and ward stay were uneventful, and the patient was discharged 6 days postoperatively with chest radiography that showed a small cavity in the RLZ.

**Fig. 3:**
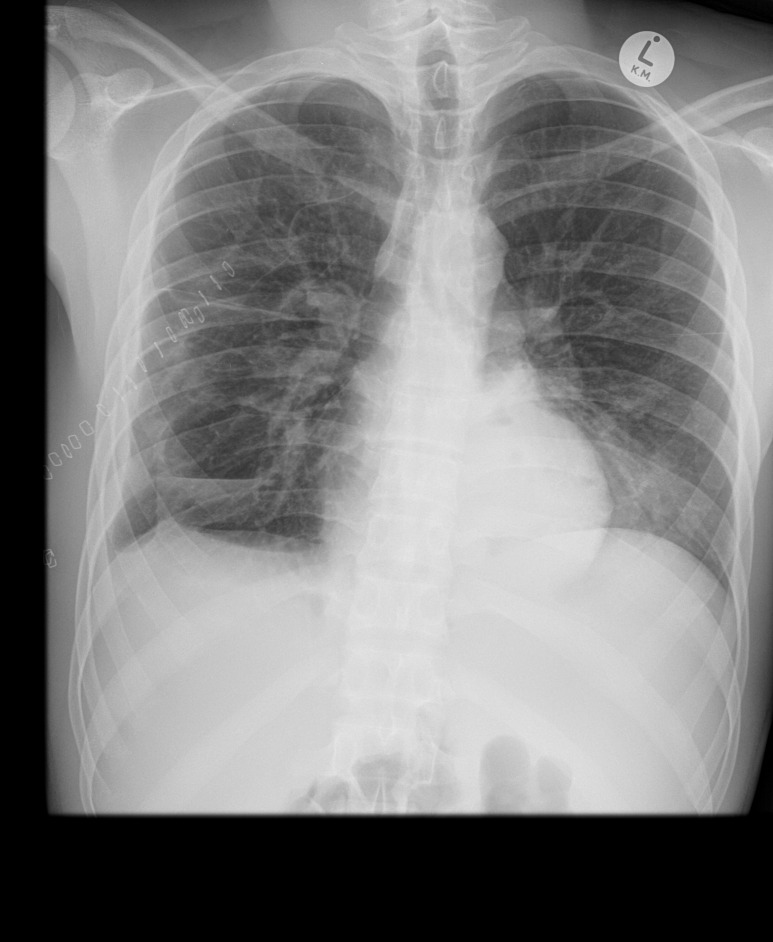
Chest radiograph on day 8 post right thoracotomy

The patient presented again to the emergency department 8 days after discharge with another episode of significant haemoptysis. The repeat chest radiograph showed a small cavity in the right hemithorax with a fluid level suggestive of the cystectomy space and a near normal looking left hemithorax (Fig. 3). He was admitted and had medical management of haemoptysis. He then experienced another episode of massive haemoptysis, this time with airway compromise evidenced by CO_2_ retention on the arterial blood gas (ABG). The repeat chest radiography showed expansion of the right-sided cavity and a left lower zone opacity. At this time, the working diagnosis was bleeding on the operated side and aspiration to the left chest. Bronchoscopy at this time showed blood in both main bronchi. Emergency right thoracotomy was then performed where few air leaks were closed and the cavity was capitonnaged. There was no obvious blood noted within the cystectomy cavity though minimal blood remained in the pleural cavity. The patient was extubated after routine drain placement and chest closure.

**Fig. 4:**
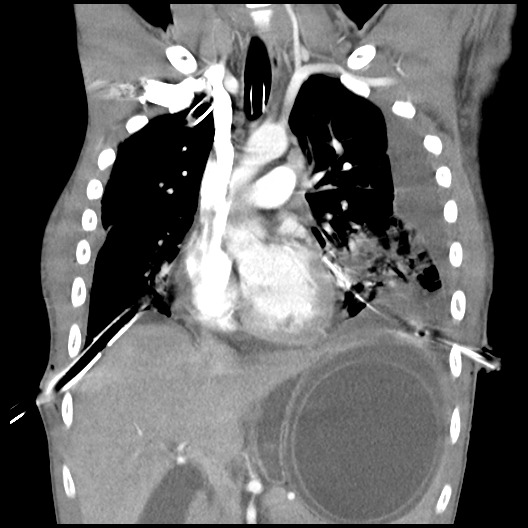
Repeat computed tomography chest scan showing intact splenic cyst and left effusion.

He developed another significant bout of haemoptysis which required reintubation 24 hours later. A post intubation chest radiograph showed significant pleural collection and a chest drain was placed to drain the space. An urgent CT chest scan showed a small left lower lobe cavity with surrounding necrotic features, left pleural collection with a collapsed and consolidated left lower lobe. The left splenic cyst was noted to be intact (Fig. 4).

Emergency bronchoscopy and left thoracotomy were performed on the patient. Bronchoscopy showed a significant blood clot in the left main bronchus and clear right main bronchus. Left thoracotomy revealed a complicated cyst in the left lower lobe with significant consolidation of the left lower lobe and the cavity was noted to be small with foul smelling blood clots. A pulsatile bleeding vessel noted within the cavity wall was ligated with 3/0 vicryl and the cavity was capitonnaged. After routine placement of two chest drains, chest closure was followed by an uneventful ICU stay

**Fig. 5:**
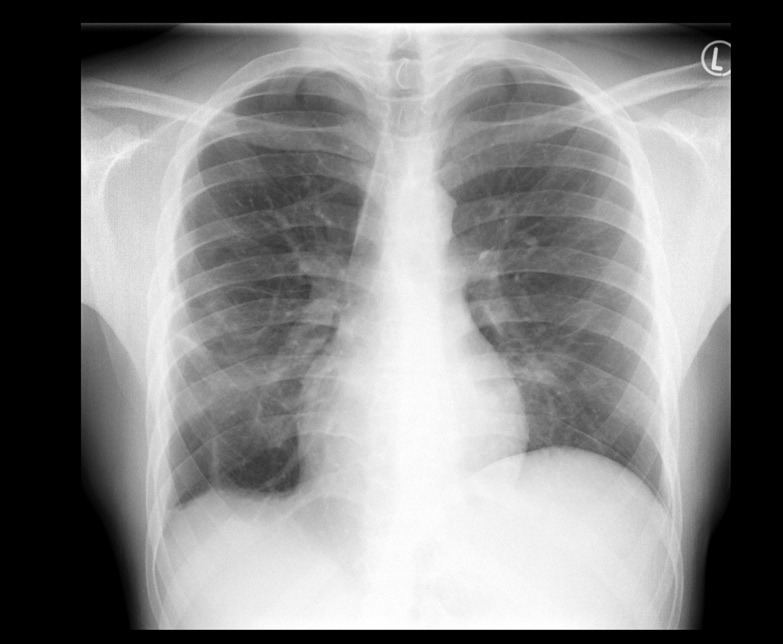
Chest radiograph 2 months post bilateral thoracotomy

He recuperated well in the ward and was discharged home after 5 days and no further episode of haemoptysis was reported. The latest chest radiograph is shown in (Fig. 5). The patient is currently doing well clinically and is scheduled for yearly review with a chest radiograph to exclude recurrence. He was also referred to our general surgical team to manage his splenic cyst.

## Discussion

Rupture of a lung hydatid cyst can be on the basis of spontaneous, iatrogenic or traumatic cause. The rupture can occur into the pleural or pericardial cavity as well as the sub-diaphragmatic space. Coughing, dyspnoea, fever, haemoptysis, flank pain, chest pain and secondary pneumonia with pressure to the neighbouring bronchi can develop due to the pulmonary rupture of the hydatid cyst on the diaphragmatic surface of the liver.^[3]^

Haemoptysis in adults is most often caused by tuberculosis, bronchiectasis and trauma or bronchogenic carcinoma – parasitic aetiology is very rare. The mechanism of haemoptysis may be due to pressure erosion of a bronchus or an obstructive effect with bronchial infection. Hydatid cysts of the lungs are commonly solitary, well circumscribed and unruptured and usually occur at lung bases.^[4,5]^

This paper reports on a patient who presented with significant haemoptysis secondary to a complicated hydatid cyst. He was also found to have bilateral cystic lesions with the right cyst uncomplicated. Most patients who present with haemoptysis are commonly found to have active pulmonary tuberculosis (TB) or sequelae of previous pulmonary TB. This case report presents one of the rare causes of haemoptysis and presents a diagnostic dilemma to a treating physician. He was initially treated with anti-tuberculosis therapy but later found to have bilateral hydatid cysts of the chest with no liver involvement.

Haemoptysis in this patient had resolved by the time he was referred for surgical intervention, which was 1 week after presentation to casualty. The decision to offer an elective procedure was then made where an appointment was scheduled for the patient within 4 weeks. The repeat chest X-ray showed near complete resolution of the cyst on the initially complicated left cyst and he was then offered a right thoracotomy to excise the large right-sided cyst. Upon full recovery after initial surgery, the patient would be planned for future resection of his left-sided cyst. This patient represented with massive haemoptysis within a week after being discharged. The cause of his haemoptysis was difficult to diagnose and radiological imaging and bronchoscopy were unhelpful in identification of the source of index haemoptysis episode. We re-opened the thoracotomy site to look for the source and found none. He was readmitted to ICU, where he experienced another bout of significant haemoptysis. At this stage, there was radiological deterioration on the left side and an urgent left thoracotomy was performed, which lead to resolution of his haemoptysis.

This case presents a challenge from a diagnostic perspective in terms of establishing the aetiology of haemoptysis in the setting of a high prevalence of tuberculosis. It also presented a surgical challenge as to which site to operate on initially, i.e. small complicated cyst v. large uncomplicated cyst, which carries a risk of rupture during surgical manipulation if the contralateral site is operated first.

## Conclusion

This case report suggests a high index of suspicion for pulmonary hydatid cyst as a cause of haemoptysis in a patient who comes from a sheep-rearing community, despite the fact that tuberculosis is the most common cause. It also suggests that one should consider operating on the complicated side first, if haemoptysis was the initial presenting complaint of the patient, despite the size of the uncomplicated contralateral site.
